# Septins Focus Cellular Growth for Host Infection by Pathogenic Fungi

**DOI:** 10.3389/fcell.2017.00033

**Published:** 2017-04-05

**Authors:** Michelle Momany, Nicholas J. Talbot

**Affiliations:** ^1^Department of Plant Biology, University of GeorgiaAthens, OH, USA; ^2^School of Biosciences, University of ExeterExeter, UK

**Keywords:** septins, filamentous fungi, branch, germ tube, invasion, pathogenesis

## Abstract

One of the key challenges faced by microbial pathogens is invasion of host tissue. Fungal pathogens adopt a number of distinct strategies to overcome host cell defenses, including the development of specialized infection structures, the secretion of proteins that manipulate host responses or cellular organization, and the ability to facilitate their own uptake by phagocytic mechanisms. Key to many of these adaptations is the considerable morphogenetic plasticity displayed by pathogenic species. Fungal pathogens can, for example, shift their growth habit between non-polarized spores, or yeast-like cells, and highly polarized hyphal filaments. These polarized filaments can then elaborate differentiated cells, specialized to breach host barriers. Septins play fundamental roles in the ability of diverse fungi to undergo shape changes and organize the F-actin cytoskeleton to facilitate invasive growth. As a consequence, septins are increasingly implicated in fungal pathogenesis, with many septin mutants displaying impairment in their ability to cause diseases of both plants and animals. In this mini-review, we show that a common feature of septin mutants is the emergence of extra polar outgrowths during morphological transitions, such as emergence of germ tubes from conidia or branches from hyphae. We propose that because septins detect and stabilize membrane curvature, they prevent extra polar outgrowths and thereby focus fungal invasive force, allowing substrate invasion.

## Introduction

Fungi are responsible for many of the world's most serious diseases, ranging from crop diseases—which each year severely restrict the global harvest of many of our most important food sources—to human diseases that claim many hundreds of thousands of lives annually (Brown et al., 2012; Fisher et al., 2016). Understanding fungal pathogens is therefore vital from economic, social, and humanitarian standpoints. There is an urgent need for more durable resistance to crop pathogens and more effective drugs to treat human diseases. Fungal pathogens share many conserved features, irrespective of the hosts they have evolved to infect, and these offer insight into the origins of fungal pathogenesis and the most likely targets for intervention and broad spectrum control of fungal disease. One of the most basic features of fungal pathogens is their ability to undergo shape changes, in order to traverse the outer barriers to infection, and gain entry to underlying tissue, which they can invade and colonize with great efficiency.

Fungi can grow as non-polarized yeast-like cells that are well-suited for moving through aqueous environments like blood in an animal host, or water on a leaf surface. Many fungi also resist environmental stress by forming non-polarized dormant resting bodies, such as sclerotia, and a huge assortment of spores to transmit pathogens long distances in search of new prey (Emmett and Parbery, 1975; Mendgen et al., 1996; Tucker and Talbot, 2001; Wang and Lin, 2012). Non-polarized fungal cells can switch to highly polarized filamentous growth, ideal for invading a heterogeneous environment, such as the tissue of an animal organ or the spaces between plant cells (Nemecek et al., 2006). This basic dimorphic ability has been further developed by pathogenic fungi that differentiate an array of specific infection cell types from fungal hyphae (Mendgen and Deising, 1993). Fungi can, for instance, form appressoria that develop enormous invasive forces—sufficient to break plastic surfaces in the laboratory—which they use to breach the tough outer cuticle of plants, rupturing epidermal cell walls to penetrate plant cells directly. This is exemplified by the rice blast fungus, *Magnaporthe oryzae*, which can generate turgor of up to 8.0 MPa (80 atmospheres) that is applied at the leaf surface to break the strong and resilient rice cuticle that coats rice leaves and stems (Wilson and Talbot, 2009). Appressoria can, however, also be used by insect pathogens to launch a huge proteolytic attack on hosts, to facilitate entry to insect bodies, where pathogens such as *Metarhizium anisopliae*, can rapidly colonize and immobilize their hosts. (Hajek and St. Leger, 1994; Zhang et al., 2010) Root pathogens, meanwhile, differentiate lobed hyphopodia from hyphae to infect root tissue. Fungal pathogens, in fact, can form a plethora of infection structures, such as compound appressoria and infection cushions (Mendgen et al., 1996). This ability to change shape and switch rapidly between non-polarized and polarized cell types is therefore fundamental to fungal pathogenesis and, indeed, the fungal lifestyle more generally.

We recognize that septins play critical roles in cytokinesis, cell cycle control, spatial compartmentalization, and as ER and plasma membrane diffusion barriers. Though these roles are undoubtedly important for fungal growth and pathogenesis, they have been extensively reviewed elsewhere (Joo et al., 2005; Gladfelter, 2006, 2010; Lindsey and Momany, 2006; Lichius et al., 2011; Hernandez-Rodriguez and Momany, 2012; Mostowy and Cossart, 2012; Spiliotis and Gladfelter, 2012; Bridges and Gladfelter, 2014). In this mini-review, we focus instead on the role of septins in the morphogenetic switch from non-polar to polar growth, generally seen in filamentous fungi as the formation of polar outgrowths, and the importance of this switch for tissue invasion and fungal pathogenesis.

## Septin-dependent host infection by fungal pathogens

**Figure 1:**
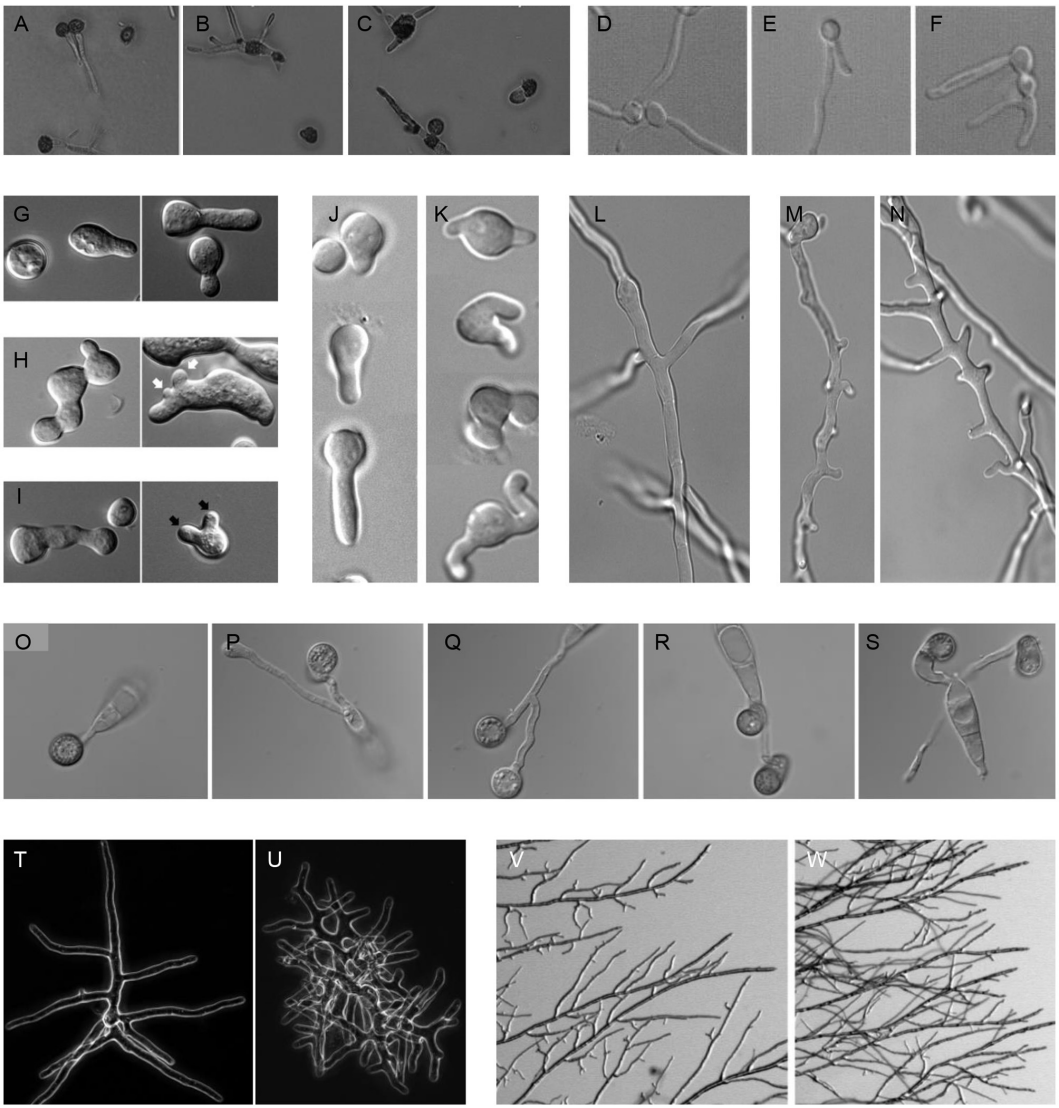
**Polar outgrowths of septin mutants. (A–C)** Germinating spores of *Ustilago maydis* incubated at 22°C **(A)** WT, **(B)** *sep1*^*cdc3*^Δ, **(C)** *sep2*^*cdc12*^Δ (Alvarez-Tabares and Perez-Martin, 2010). **(D–F)** Filaments of *Candida albicans*, **(D)** WT, **(E)** *cdc10*Δ, **(F)** *cdc11*Δ (Warenda and Konopka, 2002). **(G–I)** Germinating spores of *Neurospora crassa*, **(G)** WT, **(H)** Δ*cdc-3*, **(I)** Δ*cdc-10*. Black arrows denote multiple germ tube emergence. White arrows show multiple conidial anastomosis tube emergence (Berepiki and Read, 2013). **(J–K)** Germinating spores and **(L–N)** emerging branches of *Aspergillus nidulans*, **(J, L)** WT, **(K, M, N)** Δ*aspB*^*cdc3*^ (Hernandez-Rodriguez et al., 2012). **(O–S)** Germinating spores of *Magnaporthe oryzae*, **(O)** WT, **(P)** Δ*sep3*^*cdc3*^, **(Q)** Δ*sep4*^*cdc10*^, **(R)** Δ*sep5*^*cdc11*^, **(S)** Δ*sep6*^*cdc12*^ (Dagdas et al., 2012). **(T, U)** Branching colonies of *Ashbya gossypii*, **(T)** WT, **(U)** *Agcdc12*Δ (Helfer and Gladfelter, 2006). **(V, W)** Branching colonies of *Neurospora crassa*, **(V)** WT, **(W)** Δ*cdc-3* (Berepiki and Read, 2013).

An increasing body of work suggests that septins play important roles in fungal pathogens (Douglas et al., 2005; Bridges and Gladfelter, 2014; Khan et al., 2015; Vargas-Muniz et al., 2016). An examination of fungal septin mutants suggests that a critical septin role is defining and restricting polar outgrowths that allow the fungus to invade and explore host tissue. In fungal pathogens of plants and insects these polar outgrowths often elaborate specialized structures to penetrate a protective barrier. Strikingly, in most fungal pathogens deletion of septin-encoding genes leads to an increase in polar outgrowths and a decrease in virulence. In the few cases where deletion of septins does not result in increased polar outgrowths, virulence is not reduced (Table 1 and Figure 1). In the rice blast fungus *M. oryzae*, for example, it has been shown that the invasive appressorium requires septins to function (Dagdas et al., 2012). A hetero-oligomeric septin ring is involved at the initial point of appressorium differentiation, forming at the base of the fungal germ tube. Septin deletion mutants often bifurcate at this point forming two germ tubes and appressoria (Figures 1O–S). Later, however, during appressorium maturation a hetero-oligomeric septin ring forms at the base of the appressorium, once it reaches a critical turgor threshold. The septin ring scaffolds F-actin, re-modeling this into a toroidal network at the appressorium pore—a specialized zone where the cell re-polarizes to form a rigid penetration hypha to rupture the rice cuticle. The septin ring also acts as a lateral diffusion barrier, holding in place polarity determinants, such as Cdc42, the actin polymerization machinery, such as Las17 (part of the Arp2/3 complex), and BAR proteins involved in membrane curvature generation, as well as endocytosis and exocytosis. In this way, the appressorium pore acts as the specialized frontier; the point at which the host-pathogen interface is first established and a complex signaling hub in the fungal cell (Yan and Talbot, 2016). Many processes are coordinated at this zone, including the generation of protrusive actin-driven force, associated membrane curvature generation, membrane biogenesis, and cell wall biosynthesis, collectively coordinated to focus new anisotropic growth at a single point where the enormous invasive force is deployed. The septin complex forms once the appressorium reaches a threshold of turgor pressure, and requires the regulated synthesis of reactive oxygen species, catalyzed by the Nox2 NADPH oxidase complex, which is necessary for plant infection (Ryder et al., 2013). Regulated ROS may directly play a role in F-actin polymerization, while also being necessary for recruiting other polarization factors, such as Chm1 and Tea1, to the pore (Dagdas et al., 2012).

**Table 1:** **Polar outgrowths and virulence phenotypes of fungal septin mutants**.

**Fungus**	**Mutant**	**Extra/altered Polar outgrowths^a^?**	**Virulence^b^**	**References**
*Ashbya gossypii*	*Aghsl1Δ Agcdc12Δ*	Yes (BR, authors call “kinked hyphae”)	NA	Helfer and Gladfelter, 2006
*Aspergillus fumigatus*	Δ*aspA^*cdc11*^* Δ*aspB^*cdc3*^* Δ*aspC^*cdc12*^*	No	Hypervirulent in *Galleria mellonell*	Vargas-Muniz et al., 2015
	Δ*aspB^*cdc3*^*		Virulent in mice	
	Δ*aspD^*cdc10*^* Δ*aspE*		Virulent in *G. mellonella*	
*Aspergillus nidulans*	Δ*aspA^*cdc11*^*Δ*aspB^*cdc3*^* Δ*aspC^*cdc12*^*	Yes (GT and BR)	NA	Lindsey et al., 2010; Hernandez-Rodriguez et al., 2012, 2014
	Δ*aspD^*cdc10*^* Δ*aspE*	No		
*Candida albicans*	*cdc10Δ cdc11Δ*	Yes (FIL clusters)	Reduced virulence in mice	Warenda and Konopka, 2002; Warenda et al., 2003
*Cryptococcus neoformans*	*cdc3Δ cdc12Δ*	Yes, Clamp cells emerge, but do not fuse, aberrant protrusions from basidia	Reduced virulence in *Galleria*	Kozubowski and Heitman, 2010
	*cdc11Δ cdc10Δ*		NA	
*Fusarium graminearum*	Δ*Fgcdc3 ΔFgcdc11 ΔFgcdc12*	Yes (Extra spore from footcell)	Reduced virulence on wheat	Chen et al., 2016
	Δ*Fgcdc10*	No	Virulent on wheat	
*Magnaporthe oryzae*	Δ*sep3^*cdc3*^ Δsep4^*cdc10*^ Δsep5^*cdc11*^ Δsep6^*cdc12*^*	Yes (GT with terminal appressorium)	Avirulent on rice	Dagdas et al., 2012
*Neurospora crassa*	Δ*cdc-3 Δcdc-11 Δcdc-12 Δcdc-10*	Yes (GT and BR)	NA	Berepiki and Read, 2013
*Ustilago maydis*	*sep1^*cdc3*^Δ sep2^*cdc12*^Δ sep3^*cdc11*^Δ sep4^*cdc10*^Δ*	Yes (GT and FIL)	Reduced virulence on corn	Alvarez-Tabares and Perez-Martin, 2010

a *Abnormal polar outgrowths observed along hyphae, perpendicular to growth axis, relative to WT. GT, germ tube emergence from spore; BR, branch emergence from hypha; FIL filament emergence from hypha. Septation and nuclear phenotypes not shown*.

b *Virulence relative to WT controls*.

A further example of the significance of septins in host infection is provided by the wheat head blight pathogen, *Fusarium graminerarum*, where the core septins FgCdc3, FgCdc11, and FgCdc12 (but not FgCdc10), are necessary for fungal development and virulence. Targeted deletion of *FgCdc3, FgCdc11*, or *FgCdc12* led to defects in growth, conidiation, and morphology, with foot cells elaborating an extra polar outgrowth in the form of a bifurcated conidium. The Δ*Fgcdc3*, Δ*Fgcdc11*, and Δ*Fgcdc12* mutants also showed greatly reduced virulence on wheat. In contrast, the Δ*Fgcdc10* mutant had wild type growth, morphology, and virulence (Chen et al., 2016). In the corn smut pathogen *Ustilago maydis*, although septins are not necessary for primary plant infection, they are required for full symptom development in which tumors are formed that result in large-scale teliospore production. Septin deletion mutants were affected in these later stages of smut disease development and elaborated bipolar rather than monopolar filaments and extra germ tubes from teliospores (Figures 1A–C). These mutants also showed reduced virulence on corn (Alvarez-Tabares and Perez-Martin, 2010). In *Ashbya gossyipii*, a filamentous fungus that can causes stigmatomycosis disease on cotton, *Aghsl1*Δ or *Agcdc12*Δ septin deletion strains showed extra polar outgrowths from hyphae that the authors describe as “kinked hyphae” and which appear to be similar to hyperbranching (Helfer and Gladfelter, 2006; Figures 1T–U).

In the pleomorphic yeast human pathogen *Candida albicans*, targeted mutation of the *cdc10* and *cdc11* core septins led to clustering of filament emergence and a reduction in virulence and invasive growth capacity in mouse infections (Warenda and Konopka, 2002; Warenda et al., 2003; Figures 1D–F). In the basidiomycete human pathogen *Cryptococcus neoformans*, deletion of septin-encoding genes led to loss of fusion in protrusive clamp cells and attenuation of virulence in *Galleria* infections (Kozubowski and Heitman, 2010). By contrast, deletion of septin-encoding genes in the human pathogen *Aspergillus fumigatus* did not result in extra germ tubes or branches, though septation and conidiation were reduced (Vargas-Muniz et al., 2015). Strikingly, *A. fumigatus* Δ*aspA*^*cdc11*^, Δ*aspB*^*cdc3*^, or Δ*aspC*^*cdc12*^ mutants actually showed enhanced virulence in a *Galleria melonella* (waxmoth larva) model of infection, while Δ*aspD*^*cdc10*^ and Δ*aspE* showed wildtype virulence. The only septin deletion mutant to be tested in a mouse model of disease, however, Δ*aspB*^*cdc3*^ showed wildtype virulence.

The increased polar outgrowth phenotype is also seen in non-pathogenic filamentous fungi. In the model *Aspergillus nidulans*, Δ*aspA*^*cdc11*^, Δ*aspB*^*cdc3*^, and Δ*aspC*^*cdc12*^ strains showed a dramatic increase in germ tube and branch emergence (Lindsey et al., 2010; Hernandez-Rodriguez et al., 2012; Figures 1J–N). Similarly *Neurospora crassa* Δ*cdc-3*, Δ*cdc-11*, Δ*cdc-12*, and Δ*cdc-10* strains all made extra germ tubes and branches (Berepiki and Read, 2013; Figures 1G–I, V–W). Though these fungi are not generally considered pathogens, presumably protrusive growth is important to their ability to explore and invade the heterogeneous substrates they colonize as saprotrophs.

## How do septins focus invasive growth by fungi?

Given the roles identified for septins in fungal invasion of living hosts and non-living substrates, what is their likely function and can more general conclusions be made? The localization pattern of septin complexes at the periphery of fungal invasive cells is strikingly conserved, even in very diverse cell types. Septin rings, for instance, form at points of hyphal constriction, and at zones of new polarized growth (Berepiki and Read, 2013). Indeed, whenever new polarized outgrowths are formed, they appear to be flanked by septin assemblages that correspond to points of maximal membrane curvature (Gladfelter, 2006). This strong association points to a role for septins in sensing and stabilizing membrane curvature, consistent with recent *in vitro* studies of septins that show their ability to condition micrometer-scale membrane curvature generation. A recent important study showed how septins can act as sensors of micrometer scale plasma membrane curvature in *A. gossyppi*. Septins appear to preferentially localize to regions of positive curvature at the base of polarized outgrowths, branches from hyphae (Bridges et al., 2016). Bridges and co-workers showed that septins localize to the most highly curved regions of branches. When mixed with lipid bilayer-coated silica beads, purified hetero-oligomeric septin complexes of Cdc10, Cdc3, Cdc12, and Cdc11-SNAP adsorbed to beads of 1–3 μm in diameter. The adsorption was therefore dependent on the curvature of the beads, which corresponded with the curvature observed at the base of branch points. The detection of curvature appears to be an intrinsic characteristic of septins that enables them to act as landmarks for stabilizing and amplifying surface topologies.

This remarkable ability of septins to stabilize and amplify new topologies is critical to the capacity of fungi to focus force at a single point—a necessary prerequisite to invasive growth—and their ability to form outgrowths at other points along hyphae. During extension of penetration hyphae it might be necessary, for example, to prevent other branches from forming, thereby focusing growth along a single rigid filament, while at other times, particularly once tissue is invaded, the ability to branch extensively and in a pattern optimized to explore host tissue, is key to the most efficient occupation of a surrounding substrate. We argue that both focused invasion and efficient exploration must involve septins to regulate cortical F-actin interactions, corral proteins associated with endocytosis and exocytosis that collectively regulate membrane homoeostasis, and to specifically deploy polarity determinants that ultimately define new points of growth. This idea is supported by the striking phenotypes observed due to loss of septins in filamentous fungal pathogens described above (Table 1 and Figure 1). These include abnormal polarized outgrowths that form along hyphae perpendicular to the growth axis of the hypha, by aberrant branch patterning, or hyper-branching phenotypes, and with associated mis-regulation of the sites of septation, or spore separation. When considered along with the loss of virulence associated with these septin mutants (Warenda et al., 2003; Alvarez-Tabares and Perez-Martin, 2010; Kozubowski and Heitman, 2010; Dagdas et al., 2012; Chen et al., 2016), it seems likely that septins are critical for cell shape determination which is a fundamental characteristic of infection-associated morphogenesis.

Finally, the role of the aspE septins, the presumed ancestral septins found in filamentous fungi and certain ciliates and algae, but missing from yeasts and animals (Pan et al., 2007; Nishihama et al., 2011; Yamazaki et al., 2013), may offer a clue to how filamentous fungi have maintained a diverse septin repertoire to condition morphogenetic plasticity. Perhaps AspE-type septins facilitate unique hetero-oligomeric associations during different developmental stages. Evidence for such a role comes from the analysis of Δ*aspE* mutants in the multicellular growth stages of *A. nidulans* where higher order structures containing three core septins (AspA^*cdc11*^, AspB^*cdc3*^, and AspC^*cdc12*^) require AspE, but those containing all four core septins do not (Hernandez-Rodriguez et al., 2014). Intriguingly, AspE localizes in cortical patches lining the entire plasma membrane, a position that seems well-suited to organizing membranes for polar outgrowths.

In conclusion, septins appear pivotal to the ability of fungi to regulate their surface topologies to facilitate the invasion of diverse substrates—both non-living and, importantly in this context, living host tissue. Septins may fulfill such a role because they are able to rigidify the cortex to prevent aberrant polar outgrowth, while assembling specifically at points where branching and re-polarization are required. How such septin distribution is determined, however, remains unclear. Does it, for example depend solely on membrane curvature-dependent recruitment of septins? If so, what are the spatial co-ordinates that might determine sites of septin-dependent polarization? In this context, what is the role of BAR domain proteins, implicated in generating and sensing nanometer-scale membrane curvature? Do they initiate the process of invasive growth? And, more broadly, how is spatial organization of the hyphal cortex actually controlled? Is it a consequence of gradients of endocytotic and exocytotic activity that provide longitudinal co-ordinates along a hypha thereby facilitating septin aggregation at correct locations, or is this instead an intrinsic characteristic of septins themselves, as suggested by *in vitro* studies (Bridges and Gladfelter, 2016). It is clear that to answer such questions, the roles of septins in focused invasion and generation of cellular protrusions will need to be explored in much greater detail. To achieve this, there is, for example, a need for specific analysis of septin function by generation of conditional mutants, or by conditional inhibition of septin aggregation during the infection process. The use of gene silencing or conditional alleles of septin genes may offer the means to do this most effectively, so that septin assembly can be prevented directly at the point of primary host infection, or later during invasive growth. The dynamics of septin assembly within living fungal cells during the infection process also requires further analysis. This is, of course, challenging because it depends on the ability to conditionally manipulate septins, while at the same time carrying out live cell imaging. However, this is not beyond the scope of current methodologies, such as super-resolution microscopy, while correlative light and electron microscopy could provide ultrastructural analysis of such assemblies, followed by cryo-electron microscopy to study *in vivo* assembly dynamics in unparalleled detail. Deployment of these approaches in pathogenic fungi undergoing host infection, will allow a comprehensive testing of our hypothesis that septins prevent aberrant polar outgrowths and thereby focus fungal invasive force at appropriate points for substrate invasion and fungal pathogenesis. An exciting prospect.

## Author contributions

MM and NT synthesized source material, generated ideas and conclusions, and co-authored the mini-review.

## Funding

NT is funded by the European Research Council under the European Union's Seventh Framework Programme (FP7/2007-2013)/ERC grant agreement no. 294702 GENBLAST. MM was funded by NSF Grant IOS1051730 from the Developmental Systems Cluster.

### Conflict of interest statement

The authors declare that the research was conducted in the absence of any commercial or financial relationships that could be construed as a potential conflict of interest.
